# Confocal multiview light-sheet microscopy

**DOI:** 10.1038/ncomms9881

**Published:** 2015-11-25

**Authors:** Gustavo de Medeiros, Nils Norlin, Stefan Gunther, Marvin Albert, Laura Panavaite, Ulla-Maj Fiuza, Francesca Peri, Takashi Hiiragi, Uros Krzic, Lars Hufnagel

**Affiliations:** 1 European Molecular Biology Laboratory, Cell Biology and Biophysics Unit, Meyerhofstrasse 1, 69117, Heidelberg, Germany; 2 European Molecular Biology Laboratory, Developmental Biology Unit, Meyerhofstrasse 1, 69117, Heidelberg, Germany

## Abstract

Selective-plane illumination microscopy has proven to be a powerful imaging technique due to its unsurpassed acquisition speed and gentle optical sectioning. However, even in the case of multiview imaging techniques that illuminate and image the sample from multiple directions, light scattering inside tissues often severely impairs image contrast. Here we combine multiview light-sheet imaging with electronic confocal slit detection implemented on modern camera sensors. In addition to improved imaging quality, the electronic confocal slit detection doubles the acquisition speed in multiview setups with two opposing illumination directions allowing simultaneous dual-sided illumination. Confocal multiview light-sheet microscopy eliminates the need for specimen-specific data fusion algorithms, streamlines image post-processing, easing data handling and storage.

## Abstract (web summary)

Multiview light-sheet microscopy is a powerful tool for imaging relatively large biological samples over long periods of time, but scattering can limit image quality. Here, the authors combine multiview light-sheet imaging with electronic confocal slit detection to improve image quality, double acquisition speed and streamline data fusion.

## Graphical abstract

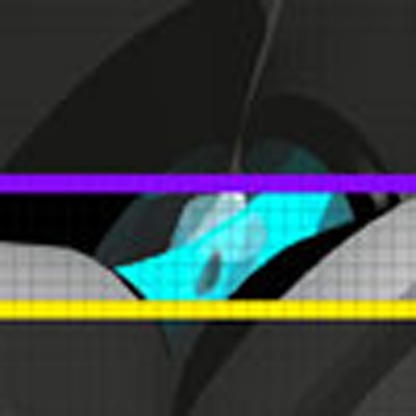

Photon propagation in biological tissues is subject to absorption and scattering^1^. To which extent these processes alter imaging can be described by a single length scale: the mean free path (MFP). Photons travelling for less than the MFP are largely unaffected by scattering (therefore having a predominant ballistic behaviour), whereas scattering dominates imaging for distances longer than the MFP. In general, the MFP varies with the biological sample and typically increases with wavelength. In fluorescence microscopy, the illumination as well as the detected light is subject to scattering. Various optical gating techniques such as time gating, confocal gating, polarization gating and coherence gating^2^,^3^ have been used to increase image contrast in three-dimensional (3D) biological tissues. These methods reduce the influence of scattered light, by shifting the distribution of detected photons towards the ballistic regime. In particular, blocking scattered light by a physical mask (pinhole or slit) or electronically directly on the detector has proven to be a simple yet powerful technique for imaging thicker specimen on confocal and light-sheet microscopes^4^,^5^,^6^,^7^,^8^.

Selective plane illumination microscopy (SPIM)^9^, characterized by orthogonal illumination with respect to detection, has gained rapid popularity due to its gentle optical sectioning capacity, which makes it a powerful tool to image thick specimens. Neglecting the wavelength dependence of the scattering process, the effect of scattering on imaging remains small if the combined illumination (*L*_*i*_) and detection (*L*_*d*_) path is shorter than the MFP (*L*_*i*_*+L*_*d*_<MFP, Fig. 1a,b). While for single objective lens microscopes illumination and detection paths are identical, the perpendicular arrangement of the illumination and detection objective lenses in light-sheet microscopy yields two independent paths (Fig. 1a). Scattering of the illumination beam degrades the quality of the light sheet away from the illumination lens and thus impairs optical sectioning. In addition, as the widefield detection (WFD) does not discriminate between scattered and ballistic photons, emitted photons from falsely illuminated regions as well as scattering of the emitted photons in general will impair the image. Typically, the regions away from both illumination and detection objective lenses yield inferior image quality.

Light-sheet setups with two opposing illumination and detection objective lenses (MuVi-SPIM, SimView, four-lens SPIM, X-SPIM)^10^,^11^,^12^,^13^,^14^ aim at yielding an *in toto* view of the sample by sequentially illuminating from two opposing directions while imaging the sample with two opposing cameras (Supplementary Fig. 4a). The acquired four views are then computationally combined to a single 3D dataset after spatial registration by fusion algorithms that assign spatially varying weights to the different views to maximize the overall image quality of the combined data set. In general, the fusion algorithms are sample and marker dependent and require adaptation as the specimen develops, changing its shape and optical properties. A straight addition of the data yields inferior results as blurred (scattered) regions are equally weighted with high-contrast regions^15^. In particular for data storage intensive experiments, light-sheet specific data post-processing poses a major computational bottleneck in the usage of multiview light-sheet microscopy.

In the following, we describe and evaluate a novel way of performing multiview light-sheet imaging through successful implementation of an electronic confocal slit detection (eCSD) mode, allowing simultaneous scanned beam illumination and detection of imaging stacks^11^,^16^. This not only doubles the imaging speed and halves the amount of acquired and stored data, but also eliminates the need for specimen-specific data fusion algorithms.

## Results

### Synthetic electronic confocal slit

**Figure 1:**
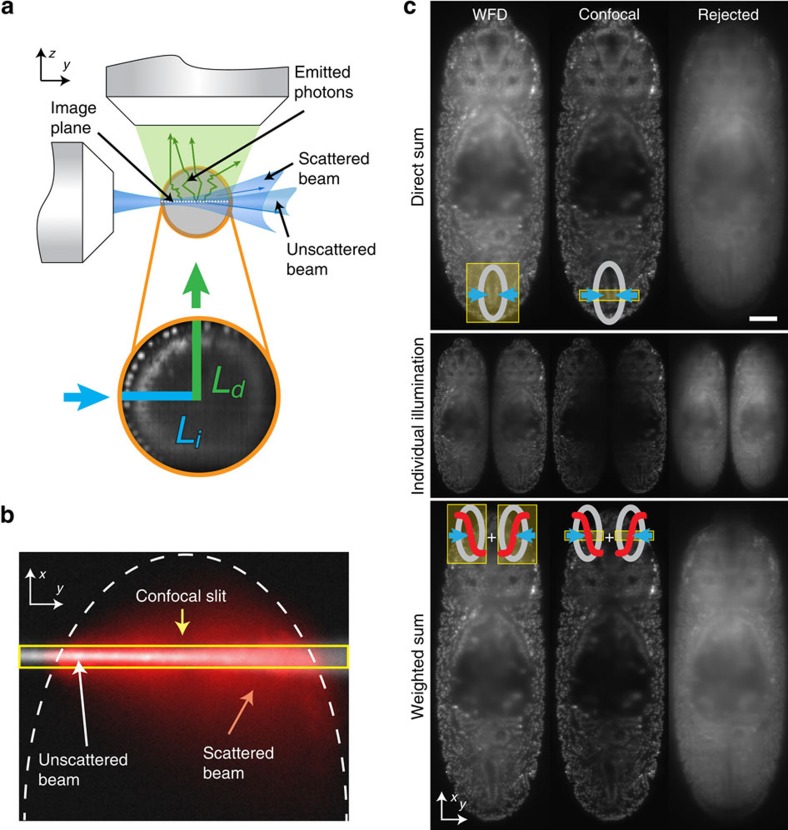
Confocal slit detection enables simultaneous dual-sided illumination with improved image quality. (**a**) Illumination (blue) and detection (green) photons in light-sheet microscopy are both independently subject to light scattering in biological specimens that lead to imaging artifacts. Image quality degrades with total length of illumination (*L*_*i*_) and detection (*L*_*d*_) paths. Combination of these data sets requires a sample dependent fusion algorithm to discriminate scattered regions. (**b**) Visualization of light scattering in a *D. melanogaster* embryo by an illumination beam entering the specimen from the left. Scattering widens the illumination beam (red) compared to the non-scattered beam (white). With the electronic confocal slit only the area inside the yellow box is recorded by the camera for the instant where the beam is located, thus discarding most of the scattered emitted light in the *x* direction (**c**) Computational analysis of walking beam images. Accumulated parked-beam positions from the two illumination directions (middle row display individual side illumination) were computationally combined either by direct addition (top row, denoted direct sum) or by sigmoidal fusion (bottom row, weighted sum). The columns display computed widefield images (left), confocal images (middle) and the rejected (scattered) light (right) for the two fusion methods. Direct and sigmoidal fusion yield similar image quality with confocal slit detection while direct fusion degrades the image under widefield detection. Scale bar is 50 μm.

In light-sheet microscopes with two opposing illumination directions (MuVi-SPIM, SimView, mSPIM, four-lens SPIM, X-SPIM and Zeiss Lightsheet Z.1), simultaneous illumination of a sample through both illumination objective lenses degrades image quality as scattered light from one light-sheet overlaps with unaltered illumination light from the other side (Fig. 1b,c). Thus, sequential illumination followed by a sample specific data fusion process has become common practice in light-sheet imaging^11^,^12^,^13^,^14^,^15^. To test whether confocal slit detection allows for a simultaneous scanned beam illumination of samples without loss in image quality, we imaged a single plane 60 μm deep inside a *Drosophila* embryo (at stage 14) expressing a fluorescent nuclear marker (His2Av-mCherry). Instead of moving the beam continuously over the sample while the camera takes a single image (which constitutes our normal imaging mode)^17^, we acquired separately for each illumination direction 2,200 images of uniformly spaced parked-beam positions covering the entire field of view (see left column in Supplementary Movie 1). This data set was then used for a detailed comparison of different illumination, detection and data fusion schemes by computationally calculating the following four images:

First, sequential widefield images with sigmoidal weighted fusion, where the parked-beam images are added separately for each illumination direction. A sigmoidal weighting function is applied to each of the two images before summing both images. Second, simultaneous widefield images (direct sum), which corresponds to the addition of all images from both illumination directions to yield a single image. Third, sequential eCSD images with sigmoidal fusion, where a confocal slit mask centred at the beam position with a fixed slit size is applied to each parked-beam image before adding all images from the same illumination direction. The resulting two images are then combined by the same sigmoidal weighting function as in the case of sequential widefield images. Finally, simultaneous eCSD images (direct sum) is analogous to the simultaneous widefield images, with the nonlinear slit mask applied to each beam position. Then all images of both illumination directions are added.

Before presenting the results of this analysis it is useful to define the slit size on the camera that corresponds to the width of the beam (1/e^2^) at the Rayleigh range. We call this scale the ‘beam-slit size', which is a dimensionless number. The beam-slit size is obtained by multiplying the size of the beam at the Rayleigh range by the detection magnification and dividing it by the camera pixel size. The reason to define the slit size at the Rayleigh range instead of at its waist directly stems from the fact that the illumination beams from both sides are offset by a Rayleigh length along the illumination direction to extend the size of the light sheet. The beam-slit size plays a similar role as the pinhole size in conventional confocal microscopy. Our optical setup allows for beam diameters ranging from 1 to 5 μm, which yields a beam-slit size of 5–27 pixels. The value of the beam-slit size divides the range of possible slit sizes in two parts: for slit sizes smaller than the beam-slit size, not only scattered light is rejected, but also non-scattered light, which results in a reduction of the overall image intensity, whereas for slit sizes much larger than the beam-slit size, the scattered light discrimination of the detection system is reduced and approaches conventional widefield imaging. We used an eCSD slit size of 1.5 times the beam-slit size in all our experiments (see below).

To evaluate the result of simultaneous illumination with eCSD we compared direct (sum) and sigmoidal fusion for computationally processed parked-beam images (Fig. 1c) for widefield and confocal detection as described above. For widefield detection, directly fused images yield inferior image quality compared with sigmoidal fusion across the whole embryo. On the other hand, sigmoidal fusion of confocal images causes a marked reduction of the image intensity along the midline of the embryo. Furthermore, only minor differences in image quality can be observed for sigmoidally or directly fused confocal images on the periphery of the embryo, where the proximal light sheet remains largely unperturbed while the distal light sheet is deteriorated by scattering, as it traverses through the entire embryo. Our computational analysis also allows for a direct visualization of the amount and spatial distribution of the light rejected by the slit mask. To this end we applied the inverse mask to each parked-beam image before adding all images (left column in Fig. 1c). As the beam traverses the specimen, scattered light accumulates and lowers the image quality (Supplementary Movie 1). The confocal detection images as calculated by sequential eCSD with sigmoidal fusion and simultaneous eCSD with direct sum methods depend on the slit size, which was set to match the beam-slit size in the above analysis. Increasing the slit size from zero interpolates smoothly between confocal and widefield image, as shown in Supplementary Movie 2. Please note, that the quality of the confocal image remains similar for a large range of slit sizes (Supplementary Fig. 5d).

### eCSD allows simultaneous dual-sided illumination

Our computational analysis showed that confocal slit detection, in addition to increasing image contrast, enables simultaneous illumination of the sample with both light-sheets without loss in image quality. To arrive at an easy to use realization of the confocal detection which does not require any additional optical components, we implemented a versatile eCSD on the latest version of a scientific complementary metal oxide semiconductor (sCMOS) imaging sensor, performing the confocal gating directly on the sensor. Based on the rolling shutter mode of the sCMOS sensor, the new control electronics allows not only to specify the number of active lines (slit size), but also the speed of the activation front of the sensor (line speed in pixels per seconds)–an essential feature to match biological requirements such as overall exposure time of the sample. Here the exposure time of the sample is given by the ratio of the total number of lines per field of view over the line speed. eCSD requires careful synchronization and spatial alignment of illumination beam positions with camera sensor activation dynamics. The required precision in timing can be estimated as follows: for a typical slit size range of 4–40 pixels, an exposure time of 20 ms and sensor size of 2,048 lines, the time for the active region to move one slit size can be as short as 40 μs. Likewise, the position of the illumination beam on the sensor needs to be controlled with an accuracy better than the slit size. The confocal slit calibration parameters need to be determined for each camera independently (see Supplementary Note 1–3 and Supplementary Figs 2 and 3 for details and Supplementary Software for a Matlab implementation to estimate the parameters from beam images in medium filled imaging chamber).

**Figure 2:**
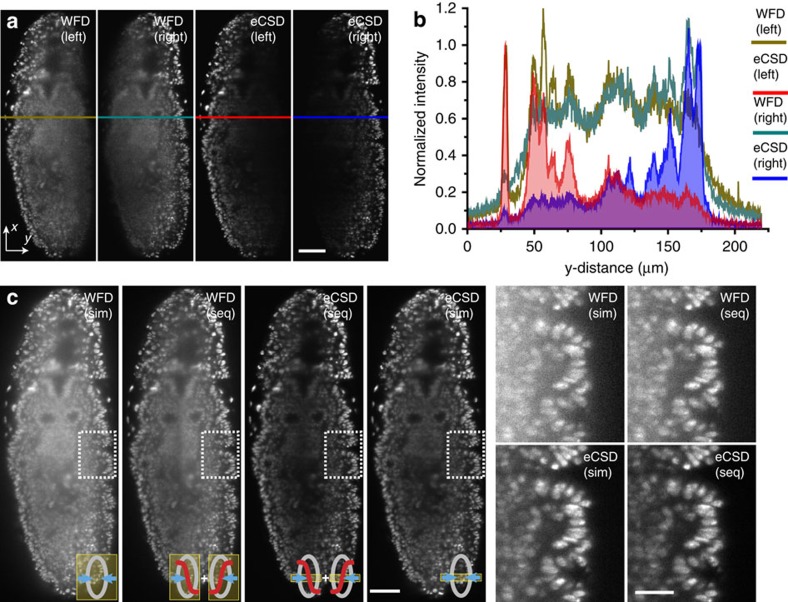
Scattered light reduction and data fusion with electronic confocal slit detection. (**a**) Left- and right-sided illuminations of a single (sagittal) plane 50 μm inside the *Drosophila* embryo expressing His2Av-RFP1 is shown for widefield and eCSD detection. The decrease in image quality away from the illumination side is clearly visible in widefield detection, while eCSD removes these blurred regions. (**b**) Intensity profiles across the embryo for indicated regions in (**a**). Intensity values close to and away from the illumination side are of equal magnitude for widefield detection (brown and green lines) preventing a direct fusion (addition) of images. eCSD profiles decay away from the illumination side as scattered photon are blocked. Please also note the removal of the scattered photon haze outside the embryo. (**c**) eCSD detection with simultaneous illumination yields superior image quality compared to the established imaging procedure of acquiring two sequential widefield images followed by a sigmoidal fusion step. Simultaneous widefield and sigmoidal fused eCSD images are shown for completeness. Magnified views of all four imaging procedures are shown on the right. Scale bars are 50 μm in both (**a**,**c**) except inserts (30 μm). For all illumination and detection schemes optical (xy) sections 50 μm deep inside an embryo at germ band retraction stage were imaged.

To evaluate the imaging and data processing benefits of eCSD for light-sheet microscopy with two opposing illumination directions, we acquired single plane images located 50 μm inside of a *Drosophila* embryo expressing a fluorescent nuclear marker (His2Av-mCherry) (see Fig. 2). We compared left and right illumination for widefield and eCSD detection respectively, along with corresponding intensity plots. While intensity levels remain mostly constant across the whole embryo for widefield detection, image quality degrades away from the illumination side (Fig. 2a,b). A simultaneous illumination yields a decrease in signal-to-noise ratio and a spatial weighting (sigmoidal) is needed to combine sequentially acquired images. This confirms the previously noted decrease in image quality for simultaneous dual-sided illumination^15^. The ellipsoidal shape of the embryo allows to dissect the influence of light-sheet and emission scattering. The influence of scattering on emitted photons originating from regions at equal distances from the midline (Supplementary Fig. 4 for orientation definitions) of the embryo is similar, as they traverse equal distances (*L*_*d*_) through embryonic tissue (neglecting tissue heterogeneities). Thus, the observed differences in image quality between regions located symmetrically to the midline are mostly due to scattering of the illumination beam resulting in a deteriorated (wider) light-sheet. The decrease in image quality from peripheral regions (proximal to the light-sheet) towards the midline, however, is due to a combination of illumination and emission light scattering, as the detection (*L*_*d*_) and illumination (*L*_*i*_) paths both increase. Compared with widefield, eCSD intensity profiles in Fig. 2b decrease in intensity as scattered photons are discarded by the detector, ending on intensities that are 5–7 times lower than the widefield counterparts approaching background levels. The efficient removal of scattered light in eCSD mode enables simultaneous dual-sided illumination without loss in image quality (Fig. 2c and Supplementary Fig. 1). Supporting contrast calculations are shown in Supplementary Fig. 5a,b. Please note, that an additional sigmoidal weighting of the eCSD images would yield detrimental results as the intensity in the middle of the embryo is further reduced. Confocal slit detection with simultaneous dual illumination thus allows acquiring complete one-sided view of a specimen without the need for data fusion or trade-off in image quality. eCSD thus halves the amount of acquired images needed, which in turn leads to an increase in imaging speed (Supplementary Note 1). Furthermore a change in slit size does imply different contrast values (Supplementary Movie 3, Supplementary Fig. 5d), and an optimum region at around 1.5 times the beam-slit size has been observed.

### Direct fusion of opposing stacks

**Figure 3:**
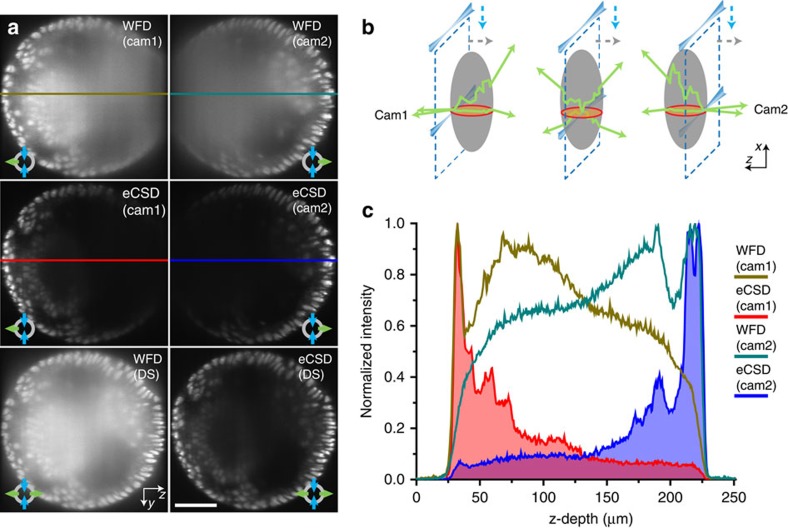
Confocal slit detection enables direct fusion of opposing views. (**a**) Transverse planes of 3D data stacks halfway between the anterior and posterior pole of an embryo expressing His2Av-mCherry (see red circle in (**b**)) are shown for widefield and eCSD detection. Top two rows show individual stacks acquired by the two opposing cameras in widefield and eCSD mode. Bottom row compares simultaneous widefield and eCSD images. Camera 1 and Camera 2 are oriented to the left and to the right, respectively. (**b**) Similar to the illumination light, also the emitted light is subject to scattering. The effect of scattering increases the deeper the image plane lies inside the embryo (dashed blue lines illustrate the light-sheet plane). The red circle indicates the transverse plane depicted in (**a**). (**c**) Comparison of intensity profile for widefield and eCSD 3D data stacks. Widefield detection collects a significant amount of photons from the far side of the embryo (around 50%), which yields a structureless blurred image (top row in (**a**)). Direct addition of the data thus results in a reduction of the signal-to-noise ratio (bottom row left panel in (**a**)). In contrast, eCSD blocks the majority of emitted photons from the far side, which enables a direct fusion (addition) of the two opposing camera stacks. All scale bars are 50 μm and all images are averages of 4 planes around the centre of the embryo.

Next we show that confocal slit detection also enables direct data fusion of opposing camera stacks. Horizontal (transverse) slices of a 3D stack of a *Drosophila* embryo acquired in widefield and confocal slit detection modes are shown in Fig. 3a for both cameras (see also Fig. 3b and Supplementary Fig. 4 for an illustration of the geometry). The degradation of the image quality with increasing depth of the light-sheet inside the embryo (away from the camera) is clearly visible and the contrast values for the projections in the last row in Fig. 3a were quantified and are presented in Supplementary Fig. 5c. Direct summation of the two stacks results in a reduced signal-to-noise ratio in widefield mode, as the scattered photons from the far side accumulate to comparable intensities as the sharp image from the other camera (illustrated by the intensity plot in Fig. 3c). On the other hand, eCSD effectively blocks scattered photons and facilitates direct summation of opposing camera stacks. Note that these results have been acquired utilizing a MuVi-SPIM setup, with two opposing scanned beam illumination directions and two opposing detection lenses. Sensor calibration parameters for both cameras need to be calculated separately as described in Supplementary Note 2. The MuVi-SPIM setup has the advantage that no sample rotation is needed to acquire full both-sided views. However, the same results apply to any multiview setup with two illumination arms with scanned beam illumination and at least one detection objective (for example, Zeiss Lightsheet Z.1 microscope). Here a 180° sample rotation is required.

### Towards a specimen independent microscope

**Figure 4:**
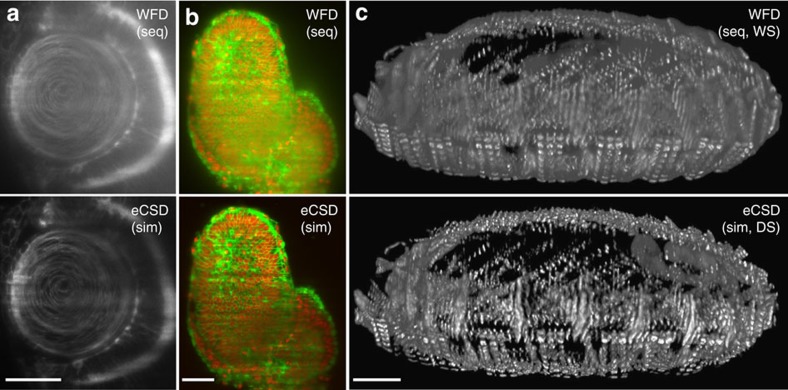
eCSD multiview light-sheet images with direct data fusion. (**a**–**c**) Examples of contrast enhancement with sequential or simultaneous illumination, direct or weighted sum by confocal versus widefield detection for different organisms and biological markers: (**a**) Zebrafish eye lens region, (Cry::CFP), (**b**) 6.5 dpf mouse embryo (R26-H2B-mCherry x mG), (**c**) 3D projections of muscle related Kettin-mCherry labeled *Drosophila* (14 h AEL). Scale bars for all subpanels are: 30 μm in (**a**) and 50μm in (**b**,**c**) and the electronic slit size was fixed to 1.5 times the beam-slit size for all eCSD images. Note that this corresponds to a slit size of 30–60 pixels on the camera sensor depending on the illumination and detection optical setup for each experiment. The illumination beam diameter was measured before the imaging to obtain the beam-slit size of the optical setup.

While *Drosophila* embryos are highly scattering and are thus well suited to illustrate the performance of confocal slit detection, we also evaluated eCSD for multiview imaging of other biological specimen (mouse, zebrafish, ascidians, plants and starfish) expressing a multitude of different biological markers. As scattering is dependent on refractive index and tissue depth changes, as well as on the wavelength and light intensity, it is important to test whether eCSD would be suited for such different conditions. Exemplifying results are shown in Fig. 4 as well as in Supplementary Movies 2,3,4,5,6 and Supplementary Fig. 8. In Fig. 4 we show three different organisms (Zebrafish, Mouse and *Drosophila*), expressing different fluorophore markers. However, for all specimen and markers we observe an increase in image quality with simultaneous illumination and direct sum of camera stacks with confocal detection compared with the widefield recordings (Supplementary Fig. 6. For further analysis on scattering effects using the mouse embryo of Fig. 4b as example see Supplementary Fig. 7.). It should be noted that for all measurements the slit size was set to 1.5 times the beam-slit size of the optical setup independent of the sample, marker and imaging depth. This allows eCSD in scanned beam multiview SPIM to be sample independent for a very wide range of different possible imaging experiments.

### Multiview-deconvolution fusion with eCSD data sets

**Figure 5:**
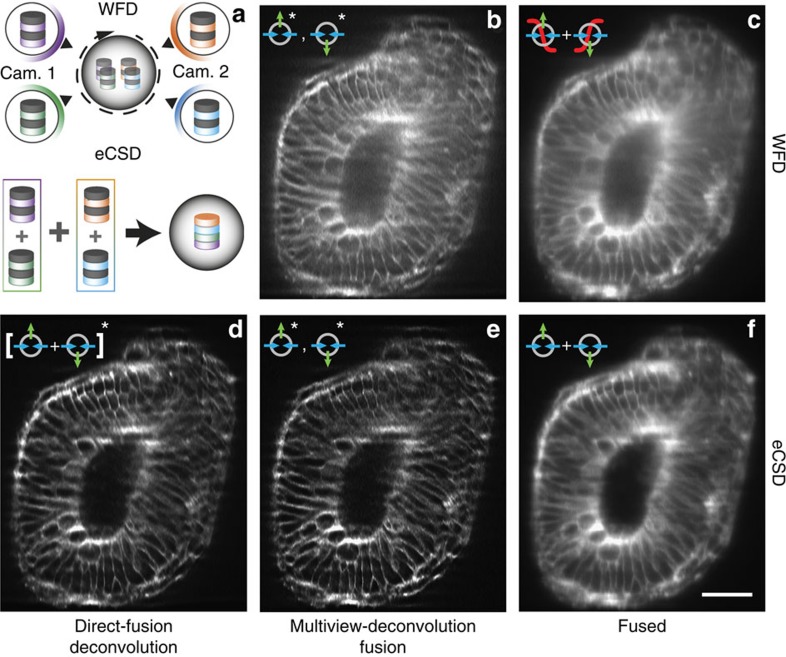
Comparison of multiview-deconvolution fusion and eCSD facilitated direct fusion. (**a**) Illustration of multiview-deconvolution fusion pipeline (top) and optimized direct-fusion deconvolution data processing for eCSD data sets. In multiview-deconvolution fusion all views (in our case 4) enter the deconvolution pipeline and are iteratively combined to a single high quality data set. In contrast the eCSD pipeline first fuses the four views to a single data set, which is then deconvolved by a classical (single view) deconvolution scheme. (**b**) Widefield multiview-deconvolution fused data set. (**c**) Sigmoidal-fused widefield data sets without deconvolution post-processing. (**d**) Direct-fused eCSD data sets followed by single view deconvolution. (**e**) Multiview-deconvolution fusion of eCSD data sets. (**f**) Direct-fused eCSD data sets without deconvolution post-processing. All subpanels display a cross section YZ-plane around 81 μm deep from the anterior side of the membrane data (mouse embryo) presented in Fig. 4b. The Fiji multiview-deconvolution plugin was used for data set shown in (**b**,**d**,**e**). Please note that for the direct-fused data set in (**d**) the Fiji plugin was used as a single view deconvolution algorithm. Supplementary Fig. 8 shows a comparison of Fiji plugin results of (**d**) with a commercial single view software package. Scale bar is 50 μm.

Next we investigate how our direct fusion approach for eCSD data sets compares to multiview data fusion by deconvolution^10^,^18^,^19^. Multiview-deconvolution fusion increases SPIM data quality post acquisition and combines multiple views imaged from different directions into a single 3D data set taking into account their respective point spread functions (PSF's). Compared with our confocal imaging scheme, where the four views (left/right illumination and two cameras) are directly added, multiview-deconvolution computationally combines the four views to a single data set by subsequent Richardson–Lucy iterations (illustrated in the upper part of Fig. 5a). The above results on direct fusion of eCSD acquired data sets suggest a streamlined pipeline for data processing as shown lower part of Fig. 5a: eCSD data sets are first fused (added) and the resulting data set is then processed by a classical (single view) deconvolution scheme. In Fig. 5 we compare multiview-deconvolution fusion results for widefield (Fig. 5b) and eCSD (Fig. 5e) data sets with deconvolved direct-fused confocal 3D data sets (Fig. 5d). For further comparison, sigmoid-fused WFD and direct-fused eCSD images are shown in Fig. 5c,f, respectively. As can be seen from Fig. 5d,e, eCSD acquired and directly fused data sets yield similar image quality as deconvolution fused confocal views and both yield superior results to corresponding widefield data sets (Supplementary Fig. 8 for a quantification). As the eCSD images were first combined to a single data set and only the resulting data set was deconvolved, this pipeline reduces both the memory requirement and computational load of the Richardson–Lucy steps by a factor four.

## Discussion

Multiview light-sheet microscopy is a powerful tool to image large specimens over extended periods of time. However, scattering can severely impair image quality and becomes limiting in many biological samples. As scattering properties change during development, data fusion algorithms for weighted sum of acquired images need to be specimen specific both in space and time, making it a costly computational operation that requires large amount of storage. We have implemented and validated eCSD on light-sheet microscopes with multiple illumination and detection directions. In addition to the previously shown improvements in image contrast^4^,^7^, we have demonstrated that eCSD allows for a simultaneous illumination of the sample from two opposing directions without loss in image quality. For scanned light-sheet microscopes with two opposing detection objectives lenses and cameras^11^,^12^,^13^,^14^, confocal slit detection also allows for direct (after 3D registration) addition of the 3D data set acquired by the two opposing cameras. Please note that systems with only one detection path require an additional 180° rotation to acquire the opposing view, but should be amenable to the same fusion pipeline. We successfully applied our eCSD to a variety of biological specimen and validated that direct fusion with eCSD yields better results than conventional WFD data fusion methods. In all experiments with either different markers or biological samples we always used a slit size of 1.5 times the theoretical beam-slit size that underlines the specimen and marker independence of our imaging and data processing approach. Our eCSD pipeline yields a doubling in acquisition speed and reduces storage requirements. As eCSD does not require any additional optical components or hardware it can be implemented on any existing light-sheet setup with the help of our calibration procedure.

We have also compared our results with deconvolution-based multiview data fusion algorithms. Our direct fusion approach to combine 3D data sets of registered opposing cameras data sets is compatible with subsequent deconvolution and yields comparable results to deconvolution fused confocal data sets. We attribute the possibility of exchange of deconvolution and fusion to the fact that both detection objective lenses have the same optical resolution and their PSF's are spatially aligned. Our direct-fusion pipeline reduces the computational workload and memory requirement by a factor four and the combined data set can be processed by classical, single view deconvolution algorithms. We would like to note that in the case of sample rotation (for example, 45 or 90°) the PSFs ‘rotate' in space and multiview-deconvolution fusion^10^,^18^,^19^ should be employed to account for this. However, also in the case of rotation our results suggest a streamlined fusion pipeline: The four data sets for each rotation setting can be directly fused to a single data set and only a single data set for each rotation position is required for the multiview-deconvolution fusion pipeline. In a case of a single 90° rotation, the number of data sets entering the multiview-deconvolution algorithm is thus reduced from 8 to 2.

In the future it would be interesting to combine eCSD with two-photon illumination to further increase image quality for highly scattering samples^20^. In addition, scanning of multiple beams together with a corresponding set of electronic masks could help to further increase imaging speed and to more evenly distribute the illumination energy for sensitive and very dynamical samples.

## Methods

### Light-sheet microscope setup

Here we briefly summarize the key components of our confocal MuVi-SPIM setup. The microscope consists of two opposing illumination and two opposing detection arms. All experiments had the following objective configuration: two Nikon 10X numerical aperture 0.3 water-dipping objective for illumination and two Nikon 25X numerical aperture 1.1 water-dipping objective lenses for detection^11^.

The main modifications compared to the MuVi-SPIM setup are a 50:50 laser beam splitter (non-polarized) to direct the laser light to both illumination objectives, tube lenses (Nikon 200 and 300 mm) to yield an effective magnification of 25X or 37.5X depending on the size of the sample.

Additionally, the sample fluorescence was imaged onto two custom modified Hamamatsu Flash 4 V1 cameras enabling confocal slit detection. These cameras are now commercially available as Hamamatsu Flash 4 V2, which include a ‘light sheet mode' based on our collaboration. A custom written script (Supplementary Software and Supplementary Note 3) calculates necessary parameters for the slit calibration. For all sample imaging we used a theoretical beam-slit size based on the 1/e^2^ size of the illumination beam.

### Hardware control

Electronic confocal slit detection requires precise timing and position control of cameras, lasers and galvanometric mirrors to ensure alignment of the illumination beam with the active area of the camera. As outlined above, we estimated the timing precision to be in the range of a few microseconds. For our optical setup, a galvanometric mirror amplitude of 1 V is sufficient to scan across the entire field of view of the camera (532 μm). A minimal slit size of 4 pixels thus yields a required precision in galvanometric mirror control voltage of 1 V*4/2048=2 mV. This can be achieved with 16-bit precision DAC. We used a custom written LabView (National Instruments) control software for synchronization of timings across all microscope devices. All trigger and analogue voltage traces are calculated by a field programmable gate array (FPGA, National Instrument NI PCIe-7842 R with a Virtex-5 FPGA) that ensures precise timing in the sub-μs range (40-MHz clock frequency). Following our collaboration with Hamamatsu Photonics, Japan, electronic confocal slit detection, also called ‘light-sheet mode', has been made available with version 2 (V2) release of the Hamamatsu Flash 4 camera. Other camera manufacturers have recently released cameras with similar features (Andor Technology, UK and PCO Imaging, Germany).

### Deconvolution

Multiview fusion deconvolution was performed with the Fiji Multiview Reconstruction plugin, (Fiji version 1.50b). For Supplementary Fig. 8, deconvolution was done with Huygens Pro with an under development SPIM module, Scientific Volume Imaging B.V, version 15.05.1p1 64b. All calculations were performed with the theoretical PSF of the optical setup.

### Zebrafish

Embryos were collected after fertilization and incubated at 28 °C. The temperature during imaging was kept constant at 23 °C. Embryos were mounted in an agarose gel (Sigma-Aldrich) inside a glass micropipette (Brand 100 μl) and a short cylinder of agarose containing the sample was then pushed out and placed in the microscope.

### *Drosophila* embryo preparation and mounting

Embryos were collected on apple juice agar plates and then dechorionated for 1 min in a fresh 50% bleach solution. Embryos were mounted in a gelrite gel (Sigma-Aldrich) inside a shortened glass micropipette (Brand 100 μl). A short segment of the gel cylinder containing the sample was pushed out of the micropipette and the pipette inserted into the microscope.

### Mouse

All animal works were performed in the animal facility at the European Molecular Biology Laboratory, according to the permission by the institutional veterinarian overseeing the operation (ARC number TH11 00 11). The animal facility is operating according to international animal welfare rules (Federation for Laboratory Animal Science Associations guidelines and recommendations).

Mouse embryos were isolated 6.5 days after plug formation by natural matings between R26-H2B-mCherry^21^ and mG (ref. 22). Embryos were dissected from the uterus and cultured in phenol red-free Dulbecco's modified Eagle's medium (Gibco, 11880-028) supplemented with 10% fetal bovine serum (PAA laboratories, A15-080), in 5% CO2 atmosphere at 37 °C and within 2 h imaged after mounting them in ultra-low melt agarose (StarLab GmbH, Germany) inside a glass micropipette similarly to zebrafish embryos.

### Ascidians

Adult *Phallusia mammillata* were acquired from the Roscoff Marine Biological Station (France). Embryo handling was done as described in Sardet *et al.*^23^. The membranes were marked with FM464 (6 μM). The embryo was imaged in artificial seawater at a temperature of 18C and mounted in the well of a 0.8% GelRite (SIGMA, G1910) plug.

## Additional information

**How to cite this article:** de Medeiros, G. *et al.* Confocal multiview light-sheet microscopy. *Nat. Commun.* 6:8881 doi: 10.1038/ncomms9881 (2015).

## Supplementary Material

Supplementary Information Supplementary Figures 1-8, Supplementary Notes 1-3 and Supplementary References

Supplementary Movie 1 Illustration of light scattering by parked beam analysis. Animation of the beam scanning through a Drosophila embryo (stage 14, anterior pole up, 60 μm from the surface) from acquired parked beam images. The red rectangle depicts shape of confocal slit used as a mask to create synthetic images. The beam data is used to illustrate the successive buildup of the widefield, confocal, and rejected light images as the beam scans over the field of view. Scale bar 50 μm.

Supplementary Movie 2 Confocal and rejected image as a function of slit size. The confocal and rejected images were calculated from the parked beam data presented in Supplementary Movie 1 with increasing slit sizes. For slit sizes below the beam-slit size of 40pixels, most of the scattered light is rejected by the confocal detection. For larger slit sizes the wide field image is approached. Scale bar 50 μm.

Supplementary Movie 3 Demonstration of confocal detection on sCMOS sensor. Automatic acquisition of a single plane inside a Drosophila embryo with increasing slit size. Here the beam-slit size is 1.5 and the total exposure time is 51ms independent of the slit size. All images are scaled to the same maximum intensity to facilitate comparison of the dynamic range. Scale bar 50 μm.

Supplementary Movie 4 Rendering of Drosophila embryo highlighting muscle structures. Comparison of widefield and confocal detection of a Drosophila embryo expressing a muscle marker (Kettin-mCherry, 14hrs AEL). Confocal detection reveals the fine structure of the sarcromeres. Scale bar 50 μm.

Supplementary Movie 5 Comparison of widefield and confocal detection of a Zebrafish eye. The movie shows consecutive z-slices (1μm spacing) through the developing eye of a Zebrafish embryo (1dpf) expressing nuclear and membrane markers (injected mRNA of H2B-GFP and Lyn-tdTomato). Scale bar 50 μm.

Supplementary Movie 6 Rendering of a mouse embryo. Comparison of widefield (left) and confocal (right) detection of a mouse embryo (6.5 days post fertilization) highlighting nuclei (H2B-mCherry) and cell membranes (mG-EGFP). Sigmoidal fusion was used to combine the widefield images from the opposing camera views, while the confocal images were directly fused. The 3D dataset was cut open at the middle of the embryo to visualize the inner morphology. Scale bar 50 μm.

Supplementary Software Supplementary Software for eCSD calibration. Parked beam images are used as input for calculation of three camera parameters necessary for confocal slit synchronization with scanning illumination beam. Example input images are provided.
