# Supplementary material for: Confocal multiview light-sheet microscopy
Source: Nat Commun. 2015 Nov 25;6:8881. doi: 10.1038/ncomms9881 (PMC4674777; doi:10.1038/ncomms9881)
Supplement: Supplementary Information — Supplementary Figures 1-8, Supplementary Notes 1-3 and Supplementary References [file ncomms9881-s1.pdf]

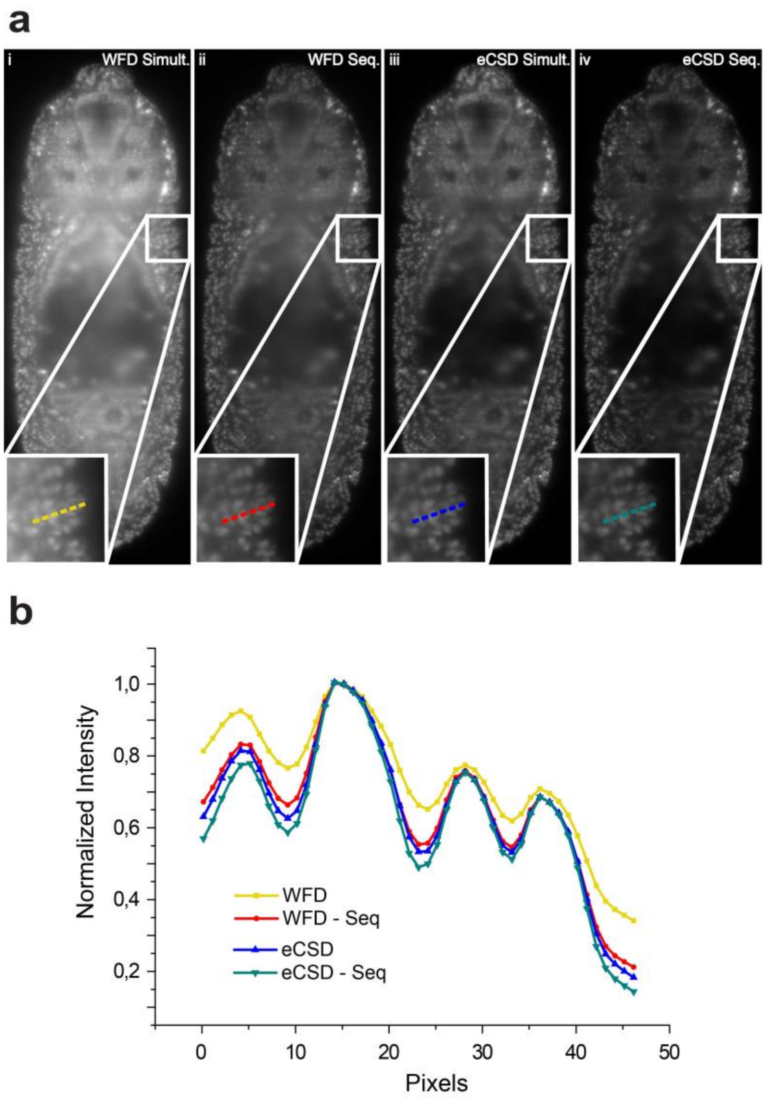

Supplementary Figure 1. Comparison of sequential illumination and simultaneous illumination for widefield detection and confocal slit. (**a**) Widefield simultaneous, widefield sequential, confocal simultaneous and confocal sequential images (from left to right) were computationally calculated from parked beam data as described in **Supplementary Note 1**. (**b**) Intensity plots along the lines depicted in (a)**.** All profiles were normalized to have a maximum intensity value of 1 to facilitate a direct comparison of the dynamic range. All images were acquired with slit size of 1.5 times the beam-slit size.

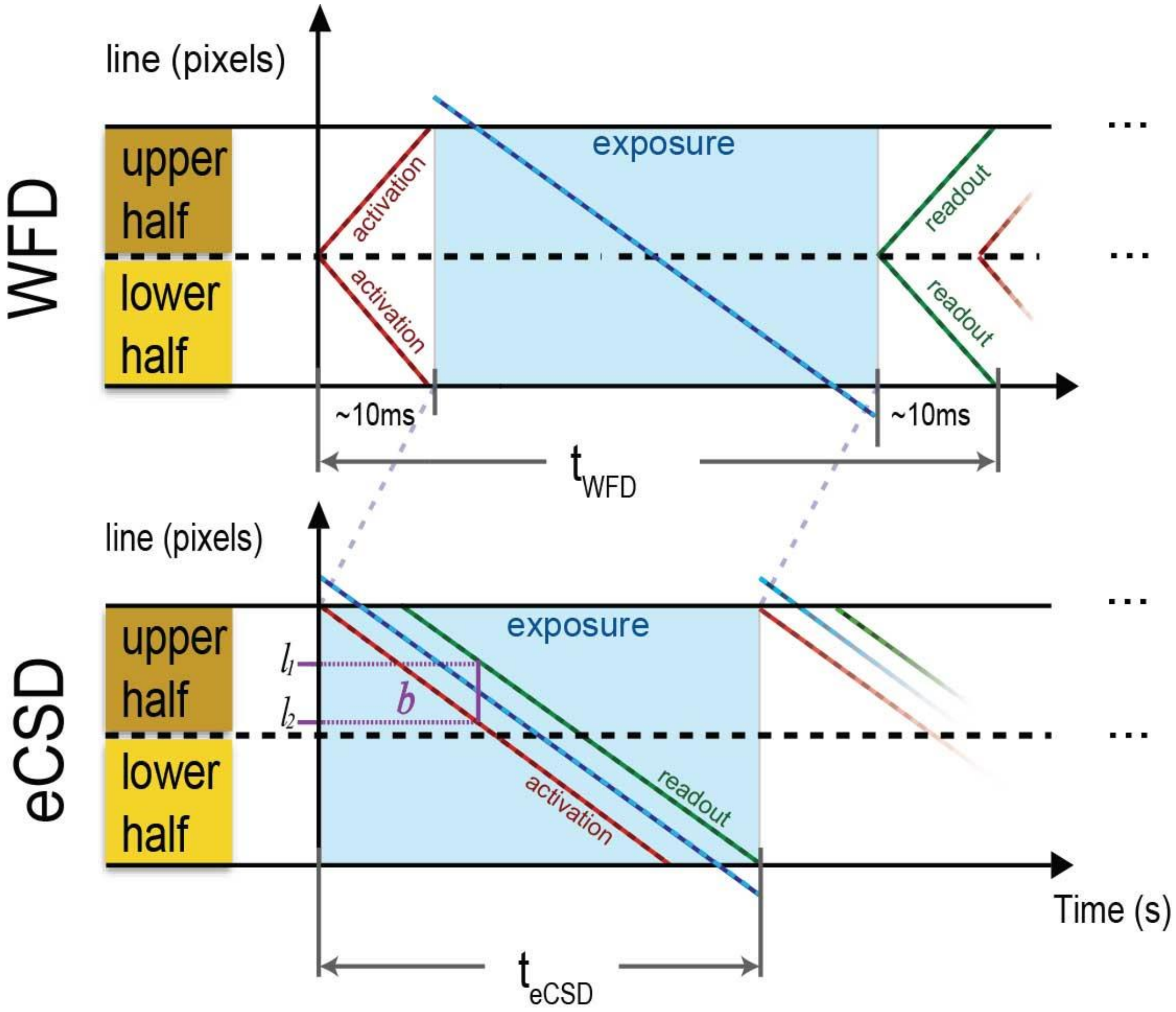

Supplementary Figure 2. Modification of the sCMOS sensor for electronic confocal slit detection. Modern sCMOS sensors are fabricated from two identical sensor halves (upper and lower). Exposure of the camera starts by an external trigger signal. However, not all lines are activated synchronously. An activation front (also called rolling shutter mode) moves over both sensor halves outwards from the middle (widefield detection). As the exposure time is the same for all lines, the readout of the pixel values (green line) follows the activation pattern after an exposure time delay. The movement of the illumination beam, in blue, starts a beam radius above and below the sensor in order to have even illumination at the edges of the image. In eCSD, the activation and readout are modified on the Gen II sCMOS camera chip. The activation direction of the upper sensor half is inverted and the lower sensor starts its regular activation pattern directly after the upper sensor is fully activated. At any time point t during the exposure only the fraction of all sensor lines between l and l' are active (illustrated by the purple line), and this is the characteristic electronic slit size b: the line at l' just starts the exposure at time t, while the line l finished accumulating photons and is read out. The image exposure is showed in light blue with the laser beam movement in time (dotted line) for the different acquisition modes.

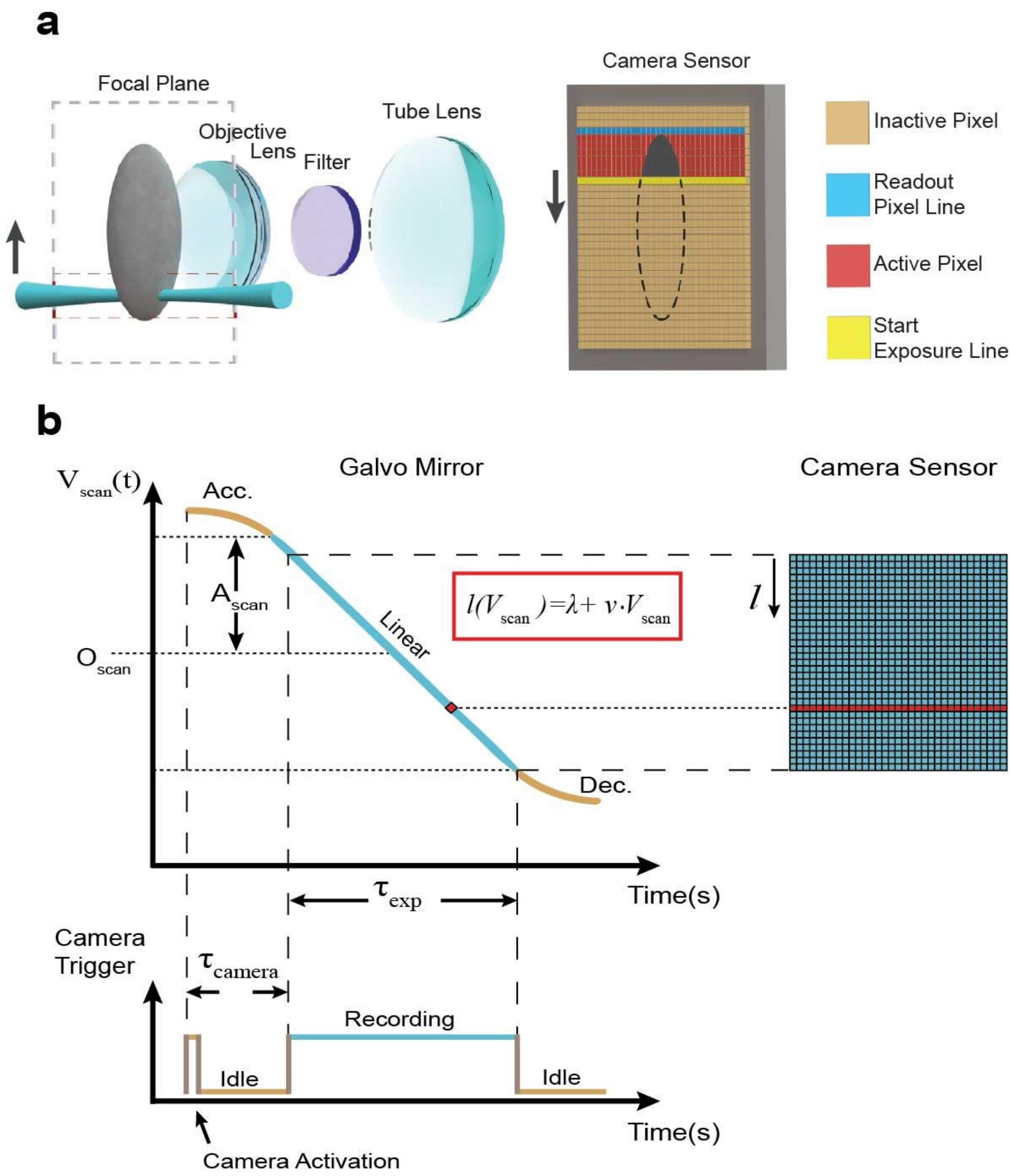

Supplementary Figure 3. Implementation of the electronic confocal slit detection for light-sheet setups. (**a**) A single detection arm of a light-sheet setup consists of an objective lens, an emission filter, a tube lens and a camera. The illumination beam moves over the sample while the camera acquires an image. For confocal slit detection, the position of the illumination beam needs to be tightly synchronized with the slit on the camera such that the center of the beam coincides with the middle of the slit mask. As the beam travels through the field-of-view, the slit follows the image of the beam on the sensor. This creates an *electronic slit* that rejects any light that falls outside of the active slit area. (**b**) Control scheme of the galvanometric mirror and mapping to camera coordinates. In between a brief acceleration and deceleration phase (red lines), the galvanometric mirror performs a linear scan of the illumination beam over the image (cyan line). At the same time trigger signals are sent to the camera and acquisition starts after delay τ_{camera}, which is needed to center the beam in the middle of the confocal slit. The galvanometric mirror is rotated back to its initial position during the readout time of the camera (not shown) to prepare for the next image.

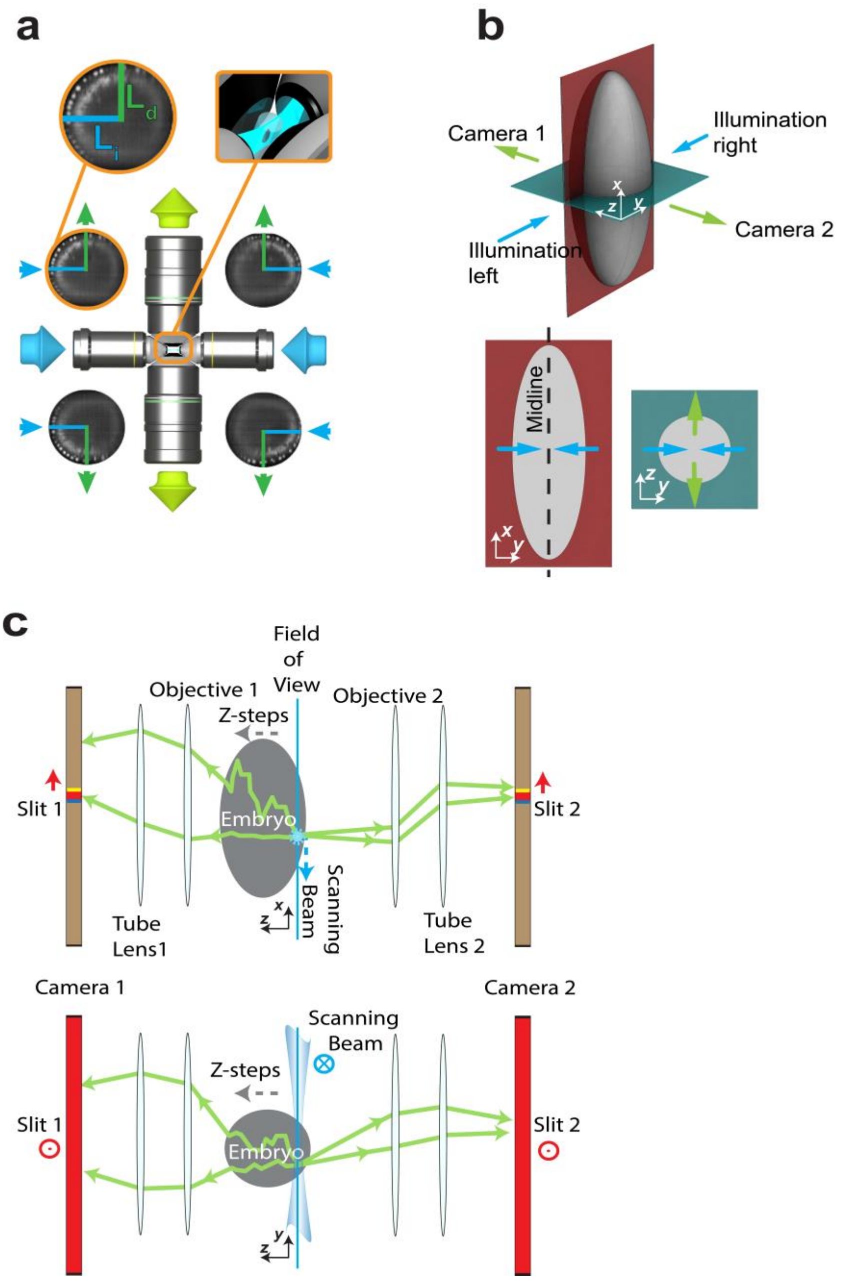

Supplementary Figure 4. Light scattering in multiview SPIM setups. (**a**) The four objective lens arrangement of the MuVi-SPIM setup allows the acquisition of four views of the sample without rotation. Transverse sections through the Drosophila embryo expressing a nuclear marker are placed between the corresponding illumination and detections objectives to show the loss in image quality due to scattering. Image quality degrades with total length of illumination (L_i) and detection (L_d) paths. Combination of these datasets requires a sample dependent fusion algorithm to discriminate scattered regions. (**b**) Representation of an ellipsoidal embryo with corresponding illumination and detection directions. The coordinate system is defined with frontal xy- plane being the illumination plane (and corresponding field of view of both cameras). The z direction is defined by the direction of stage movement through which a stack is acquired. Therefore, the transverse yz-plane is the cross section which shows all acquired images in one particular time point for all detection and illumination directions considered. (**c**) All photons that are highly scattered along the x-component are completely discarded by the confocal slit, whereas the widefield detection detects these as increase in noise, leading to blurred images. Effects of scattering only in yz plane are captured by both confocal and widefield detection modi. Depending on the slit size, scattered photons with minor components in x direction might also be detected by both detection schemes.

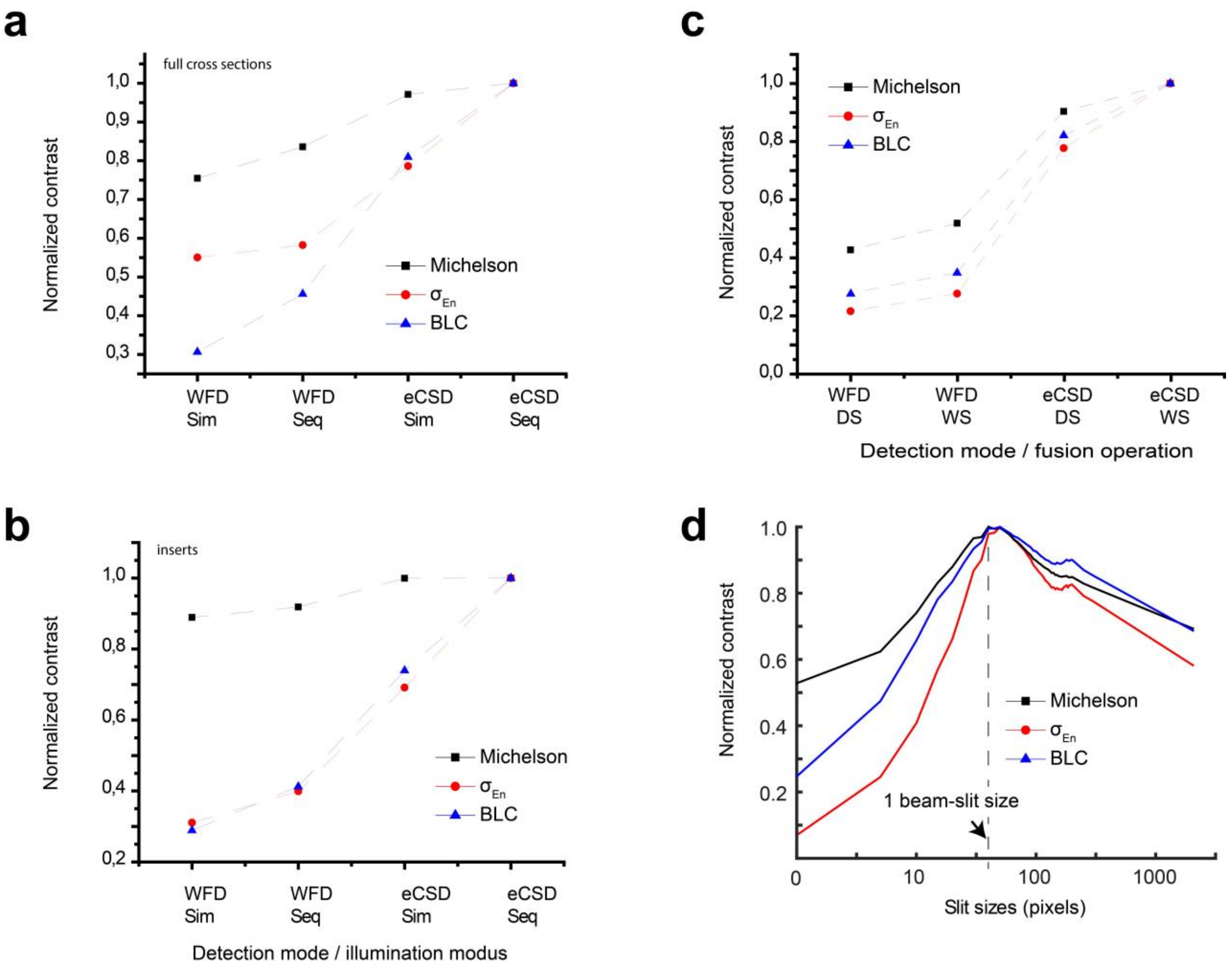

Supplementary Figure 5. **Contrast Measurements**. Quantification of image contrasts were performed by calculating Michelson contrast[5] , standard deviation of energy-normalized histograms[6] (σ_{En}) and the *Local Band-Limited Contrast*[5] (BLC, with bands defined analogous to *Useful Contrast* measures[7]), for widefield as for confocal detection comparing different fusion methods. (**a**) Contrast quantifications of the *xy*-cross section shown in **Figure 2c.** (**b**) Contrast quantifications of the *xy*-cross section shown in inserts (dashed squares) of **Figure 2c.** (**c**) Contrast quantifications of the *yz*-cross section shown in WFD (direct sum) and eCSD (direct sum) subpanels of **Figure 3a**. (**d**) Quantification of the image contrast with changing slit size of the data presented in **Supplementary Movie 3.** A maximum found around 40 pixels for a 25X detection and a 5 µm diameter illumination beam waist supports the choice of 1,5 times for beam-slit size in our eCSD acquisitions. For quantification of BLC, a frequency range corresponding from 2 µm to 20 µm was selected in both directions. Other bandwidths display similar trends.

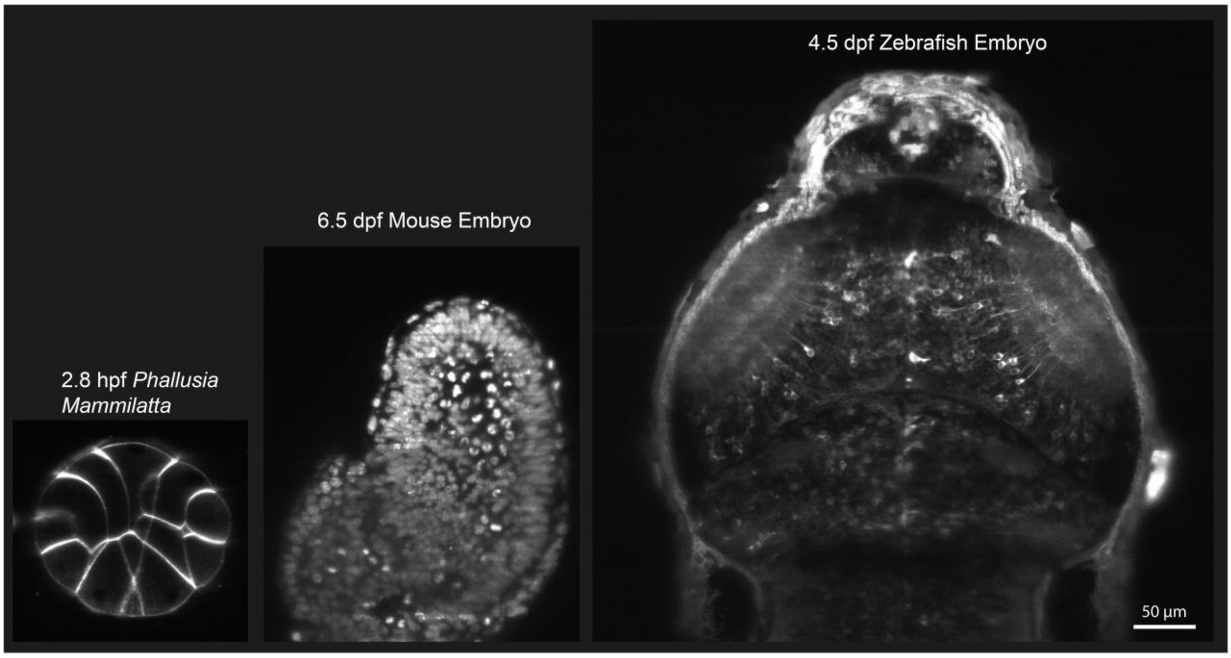

Supplementary Figure 6. Imaging specimen of different sizes. eCSD allows imaging of different samples which can range on different sizes and here we exemplify images of *Phallusia Mammilata* embryo (~140 µm), a mouse embryo (~230 µm) and a 4,5dpf Zebrafish embryo brain (~500 µm). All imaged with a fixed slit-size of 1.5 times the beam-slit size of the optical setup.

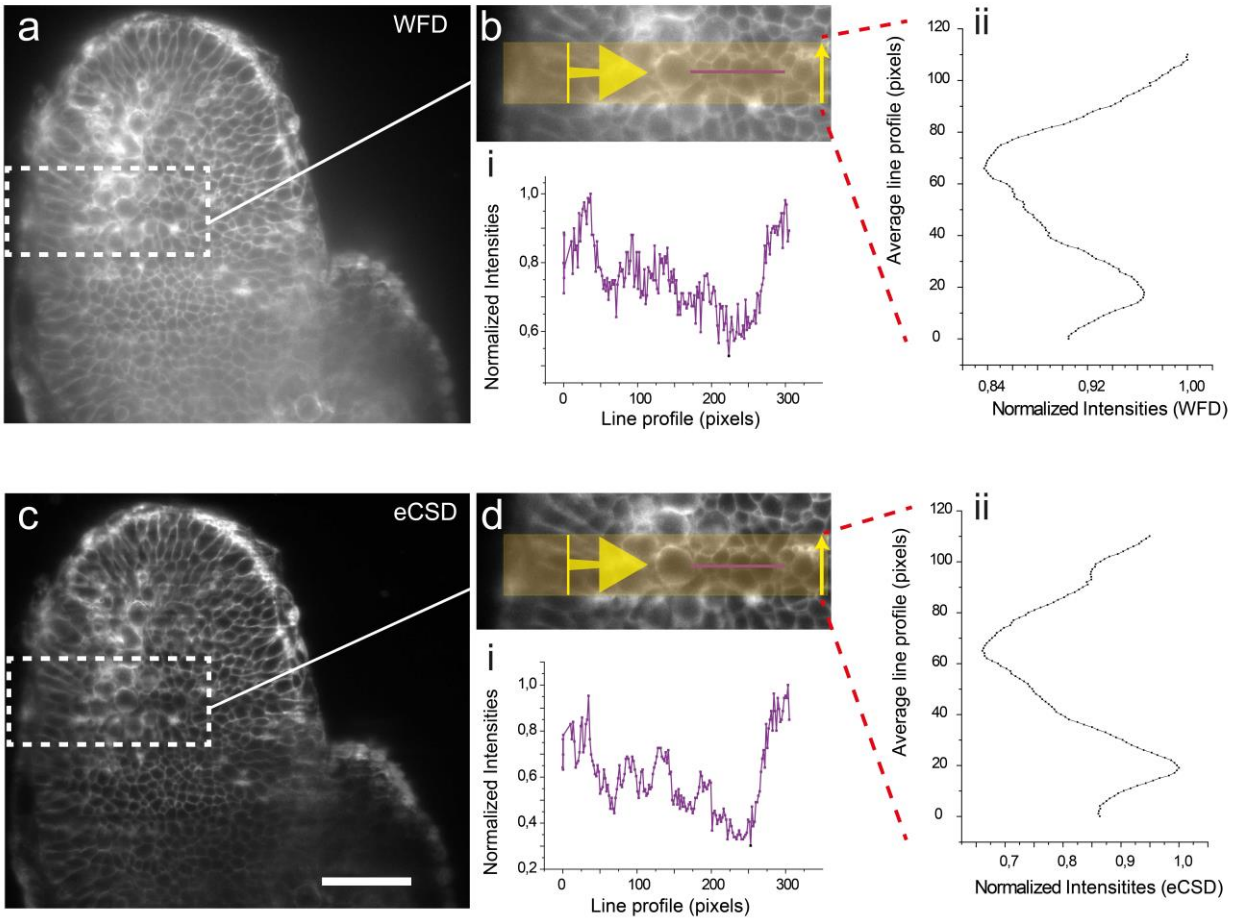

Supplementary Figure 7. Scattering effects: stripes. Due to the optical geometrical arrangement for illumination and detection paths, light sheet microscopy suffers from artifacts produced by scattering effects on the sample. Some of these effects can generate areas of shadow creating striped patterns of uneven illumination[8]. In general, scattering of the emitted light tends to mask striping artifacts. As confocal detection removes the scattered light, stripes can become more apparent. However, they are also present in the widefield image. Here we analyze the data displayed **Figure 4c**. Reproduction of WFD (**a**) and eCSD (**c**) acquired images from **Figure 4** with the same region highlighting a stripe artifact. The stripe artifacts can be noted from the marked dip in the average projection shown in (**b,d ii**) (from yellow box region, big yellow arrow represents the direction of the average projection on all columns. The average column is then plotted upwards, as indicated by smaller yellow arrow). Please note, that the dip is deeper in the confocal case, however, the signal-to-noise along the stripe (purple line) is still higher (**b,d i**). Scale bar is 50 μm.

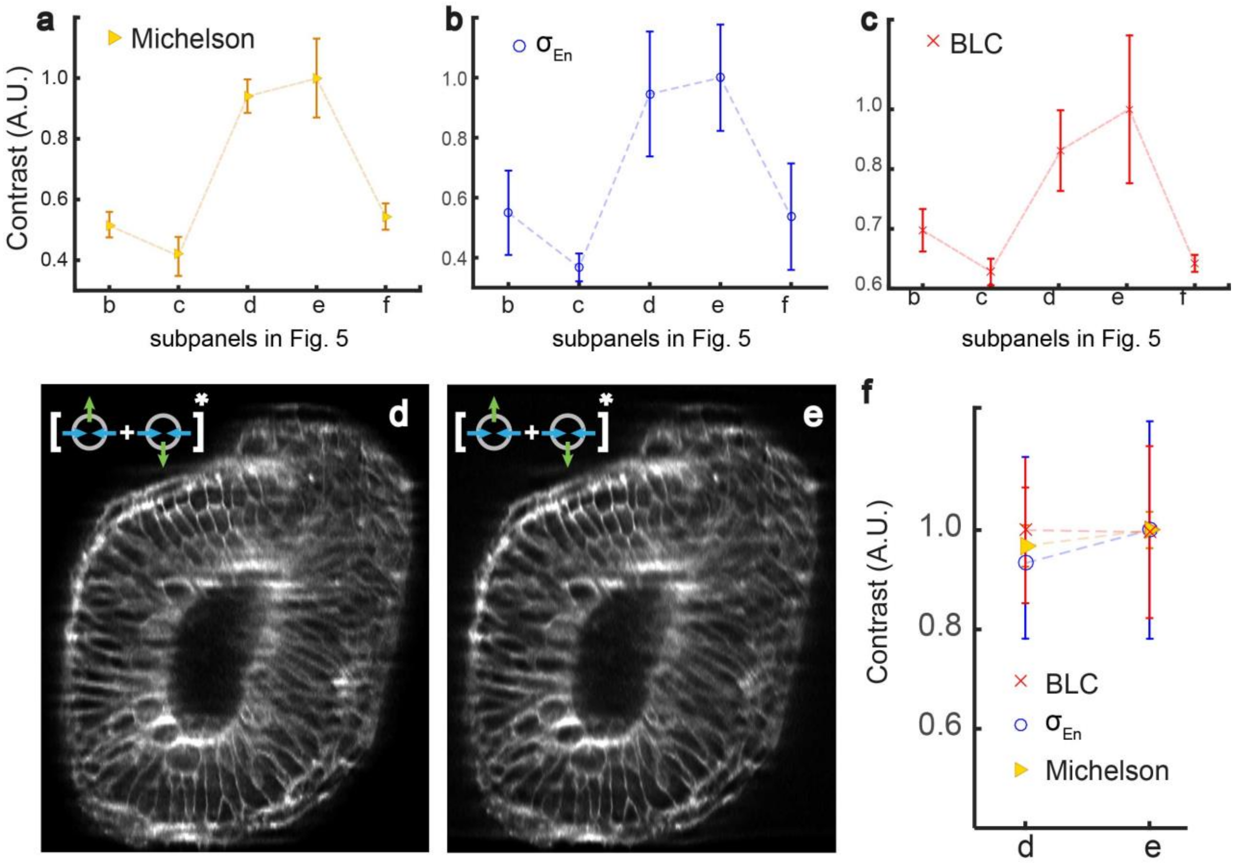

Supplementary Figure 8. Image quality quantification of multiview fusion approaches. The graphs display image quantification of the 3D dataset shown in **Fig**. **5** (main text) for (**a**) Michelson contrast[5], (**b**) energy-normalized histograms[6] (σ_{En}), and (**c**) the *Local Band-Limited Contrast*[5] (BLC, with bands defined analogous to *Useful Contrast* measures[7]) measures. We evaluated the measures in regions for 17 consecutive planes to estimate the variability across each 3D dataset. The plots show mean values and variances of each measure for widefield (sigmoidal fusion and multiview-deconvolution fusion) and confocal images (direct-fusion with deconvolution and multiview-deconvolution fusion, direct-fusion without deconvolution). The curves for each measure we scaled such that the mean of multiview-deconvolution fused datasets has a mean of unity. Multiview-deconvolution fusion and direct-fusion with deconvolution post-processing yield similar measures exceeding the image quality of widefield images. (**d,e**) Our direct-fusion approach is also compatible with "normal" (single view) deconvolution methods. Here we compare direct-fused eCSD images processed after fusion with a commercial deconvolution software package (Huygens, SVI Netherlands) in (**d**) with **Fig. 5d** of the main text repeated in (**e**). The image quality quantification in (**f**) reflects that both methods yield very similar results.

Supplementary Note 1

Implementation of electronic confocal slit detection on light-sheet setups

Confocal slit detection for light sheet microscopy has been first proposed for reduction of light coming from fluorophores excited by the side lobes of Bessel beams[1]. This method required images to be acquired each step the sample moved through a parked beam, each of the beam images are computationally masked and added to provide a full final image. It used only one illumination and one detection objective, and was slow in comparison to fast biological processes in live samples. Implementation using a physical slit with descanning optics has also been demonstrated[2]. This, however, requires extra hardware built on the detection path, and is not flexible in respect to slit size changes, for example. Later, the implementation on the chip for on illumination and one detection SPIM systems was shown, where mainly the reduction on scattered light has been characterized[3,4]. Since no work has so far been focused in systems where more than one illumination and detection directions are simultaneously considered, our work was focused on this application for live biology imaging.

As we have shown with our parked beam analysis, confocal slit detection enables simultaneous two-sided illumination without loss in image quality. Encouraged by these results we engaged in collaboration with Hamamatsu Photonics Germany and Hamamatsu Photonics Japan to modify the latest generation of sCMOS sensor (generation II) and to implement an electronic mask on the sensor. As these changes only affected the activation timing of the sensors lines (see **Supplementary Figure 2** for details), the operation mode of the camera can easily be switched between conventional area and eCSD mode by a few software commands.

In the eCSD mode of the sensor, a single activation front sweeps over the entire sensor. The activation front velocity v_{act} $[pixels \cdot s^{-1}]$ can be set in multiples of the minimal line activation time (roughly 10µs). Upon activation, the accumulated charges of the activated line are discarded and a new line-exposure starts. The duration of the line-exposure τ_{line} can be freely specified and the line is read out directly after the exposure finished. The product of line-exposure and activation front velocity yields the number of active lines on the sensor, which corresponds to the size of the confocal slit:

$$b = \tau_{\mathrm{line}} \cdot v_{\mathrm{act}}. \qquad (1)$$

The motion of the confocal slit on the camera has to be tightly synchronized (within a few micro seconds) with the position of the illumination beam such that the center of the beam coincides with the middle of the active line array (see **Supplementary Figure 3**).

Due to our collaboration with Hamamatsu Photonics now all the new Flash 4 cameras (called Flash 4 V2) have the possibility to acquire images in confocal slit modus, so called *light-sheet mode*, and are commercially available. Likewise, other camera

manufacturers have now released cameras with similar features (Andor Technology, Belfast, PCO Imaging).

A comparison of acquisition modes between rolling shutter readout and eCSD can be found in **Supplementary figure 2.** The time it takes for each sensor half either to activate or readout is 10ms. That implies that eCSD with one sensor half flipped requires twice that time before the activation front has reached all pixel rows and $\tau_{exp,eCSD}$ is 20ms or larger. In comparison between eCSD simultaneous imaging and sequential widefield imaging, Imaging with eCSD is a factor 2 faster when the condition $\tau_{exp,WFD} + 20 \geq \tau_{exp,eCSD}$ is met.

Supplementary Note 2

Calibration and synchronization of beam scanning and electronic confocal slit detection

On our MuVi-SPIM setup, a voltage controlled galvanometric mirror steers the beam over the field-of-view of the camera as depicted in **Supplementary Figure 3**. After a short acceleration phase (usually 5% of the exposure time), the laser line is switched-on and the beam moves with a constant velocity v_{beam} over the sample to ensure a uniform illumination. Synchronously with the laser activation a trigger is sent to the camera to start a new exposure after a delay τ_{camera}. Coordination of the camera with the illumination beam requires transforming the voltage coordinates that control the galvanometric mirror (amplitude A_{scan} and offset O_{scan}) and image exposure time τ_{exp} with the camera sensor settings:

$$M_b: (A_{\text{scan}}, O_{\text{scan}}, \tau_{\text{exp}}) \longrightarrow (\tau_{\text{line}}, v_{\text{act}}, \tau_{\text{camera}}). \quad (2)$$

It should be noted that this transformation also depends on the slit size b. The exact functional form of the transformation depends on the scan details. Briefly, the sensor activation velocity is only a function of the scan velocity $2A_{\text{scan}}/\tau_{\text{exp}}$ of the beam. As outlined above, the slit size b and v_{act} specify τ_{line}. The linear scan regime of the scanner starts outside of the field-of-view of the camera and the camera trigger delay accounts for the time the scan process requires to position the center of the beam at the middle of the slit. On our microscope setup, the transformation M_b is determined by the following calibration procedure:

1. The sample chamber is filled with a fluorescent dye solution to visualize the illumination beam.
2. Only one illumination beam is used to calibrate one camera first.
3. The beam is parked at different position on the sensor $(i \geqq 2)$ by applying fixed voltages V $(A_{\text{scan}} = 0, O_{\text{scan}} = \text{V})$ to the scan-control electronics. Each time, the exact center position (line index) of the beam on the camera sensor l_i is determined automatically by processing the fluorescent image.
4. Linear regression is used to relate scan-voltages to line coordinates on the sensor $(l = \lambda + \upsilon \cdot V)$. The fitting parameters λ and υ reflect the optical setup (magnification of the illumination and detection objective lens) and stay fixed, as long the optical setup remains unchanged.
5. λ and υ together with the slit size b completely determine the mapping M_b for the used detection arm.
6. This procedure is repeated for the second detection arm yielding specific fitting parameters λ' and υ' that determine the mapping M_b' for the second camera.
7. Finally, the other illumination beam is calibrated to overlap with the first illumination beam through adjustment of amplitude and offset settings.

After the calibration eCSD can be used without any further modification on the microscope. New sensor settings for both cameras are calculated by M_b and M_b' every time the galvanometer scan range, exposure time, or the slit size are modified. **Supplementary Movie 3** shows the automatic adaptation of the camera sensor settings by altering the slit size. Notably, for step 1, it is not always necessary to use a fluorescent dye. Using e.g. E3, PBS or even the residual water autoflourescence can allow a good beam characterization given sufficient laser power. Additionally, encapsulated dyes in FEP tubes could be an alternative to minimize objective staining.

Calibration settings for eCSD have been validated in combination with the Hamamatsu Hokawo software. Other camera manufactures have also implemented confocal line detection on their sCMOS models (Andor Technology Ltd., UK and PCO AG, Germany). The same calibration procedure also applies to these camera models, however the specific camera sensor settings might need to be adapted. See **Supplementary Note 3** and **Supplementary Code** for a Matlab implementation to calculate eCSD parameters.

Supplementary Note 3

Matlab implementation of eCSD calibration

Our **Supplementary Code** calculates the camera sensor settings for confocal slit detection. These settings ensure precise synchronization of the active region of the sensor with the galvanometric mirror scanning of the illumination beam. The calculation of the parameters proceeds in two steps:

Transformation of galvanometric mirror voltages to camera line positions: Our code first calculates the transformation that maps galvanometric mirror voltages to the corresponding pixel line positions of the beam on the sensor. This transformation is assumed to be linear and remains unchanged as long the optical setup is not modified. The script expects a series of images ($\geqq 2$) of parked beams saved as "tif"-files. For best fitting results it is suggested to maximize the spacing between beams. The filenames should follow the nomenclature "Calibrate_$*voltage*$.tif", where $voltage$ specifies the galvanometric mirror voltage. The script assumes horizontal beam orientation aligned with the camera sensor lines. Care should be taken to acquire a clean image of the beam in fluorescent medium to avoid fitting artifacts. To estimate the beam position on the sensor, a Gauss-fit of the side-projected intensity image is performed. The calibration needs to be performed on the non-cropped sensor.

Calculation of camera settings: The second part of the code (same Matlab-file) uses the voltage-to-pixel transformation to calculate the confocal slit sensor settings: CameraLineInterval (v_{act}), CameraDelay (τ_{camera}) and InterlineExposure (τ_{line}). It requires as an input the exposure time, the galvanometric mirror settings (amplitude, offset, and turn time percentage), and the slit-size in pixels. For a cropped sensor, one also needs to specify the topmost pixel row index.

Please note that our calibration code strictly requires galvanometric mirror voltage traces and camera triggers as illustrated in **Supplementary Fig. 3**. The camera trigger needs to coincide with the beginning of the galvanometric mirror acceleration part. As shown in **Supplementary Fig. 3**, the galvanometric mirror motion consists of three parts: acceleration (parabolic), linear and deacceleration (parabolic) part. The fraction of parabolic periods to the entire galvanometric mirror motion time specifies the turn time percentage. The camera needs to be configured in external trigger mode (edge trigger). Care should be taken that the sensor readout directions coincides with the beam scanning.

Supplementary References

1. Fahrbach, F. O. & Rohrbach, A. Propagation stability of self-reconstructing Bessel beams enables contrast-enhanced imaging in thick media. *Nat. Commun.* **3,** 632 (2012).
2. Silvestri, L., Bria, A., Sacconi, L., Iannello, G. & Pavone, F. S. Confocal light sheet microscopy: micron-scale neuroanatomy of the entire mouse brain. *Opt. Express* **20,** 20582–20598 (2012).
3. Mei, E., Fomitchov, P. a., Graves, R. & Campion, M. A line scanning confocal fluorescent microscope using a CMOS rolling shutter as an adjustable aperture. *J. Microsc.* **247,** 269–276 (2012).
4. Baumgart, E. & Kubitscheck, U. Scanned light sheet microscopy with confocal slit detection. *Opt. Express* **20,** 21805–21814 (2012).
5. Peli, E. Contrast in complex images. *JOSA A* **7,** 2032–2040 (1990).
6. Keller, P. J. *et al.* Fast, high-contrast imaging of animal development with scanned light sheet-based structured-illumination microscopy. *Nat. Methods* **7,** 637–642 (2010).
7. Truong, T. V., Supatto, W., Koos, D. S., Choi, J. M. & Fraser, S. E. Deep and fast live imaging with two-photon scanned light-sheet microscopy. *Nat. Methods* **8,** 757–760 (2011).
8. Rohrbach, A. Artifacts resulting from imaging in scattering media: a theoretical prediction. *Opt. Lett.* **34,** 3041–3043 (2009).
